# Editorial: Multisensory speech in perception and production

**DOI:** 10.3389/fnhum.2024.1380061

**Published:** 2024-02-19

**Authors:** Kauyumari Sanchez, Karl David Neergaard, James W. Dias

**Affiliations:** ^1^Department of Psychology, Cal Poly Humboldt, Arcata, CA, United States; ^2^Institute for the Future of Education Europe, Tecnologico de Monterrey, Comillas, Spain; ^3^Medical University of South Carolina, Charleston, SC, United States

**Keywords:** multisensory, speech perception, multisensory integration, cross-linguistic, audio-visual speech, trimodal speech perception

This Research Topic addresses the multisensory nature of speech by investigating contexts in which information from various sources are and are not used to facilitate speech perception. The research presented in this topic suggests that one's culture, language experience, and expectations impact one's ability to effectively use multisensory information (Zeng et al.; Zhang et al.). In addition, the utilization of a given sensory stream may vary depending on the presence and clarity of additional sensory streams in the environment (Hansmann et al.). Further, it is argued that multisensory information plays a dominant role in speech perception, as compared to lexical information (Dorsi et al.).

Zeng et al. investigate the role of sensory information (visual-only, audio-only, and audiovisual) in the perception of Mandarin lexical tone (T1, T2, T3, and T4) among native and non-native speakers. Given that the visual impact of changes in tone may be subtle, the researchers compared natural speech to clearly spoken speech productions (speech style) with the purpose of identifying category distinctions due to either signal-based cues (i.e., articulatory features such as head and eyebrow movements) or code-based cues (i.e., acoustic features such as F0). The results revealed differences across the tones for speech style and modality, indicating that clear speech benefits the perception of acoustically salient tones (i.e., Tones 1 and 4), while the perception of tones that may be visually salient (i.e., Tones 2 and 3) is benefited from the presence of visual speech. Together this indicates that code-based cues impact the acoustic and visual attributes that are present in clear speech. Signal-based cues, meanwhile, did not contribute to the perception of tones for native speakers, but did for non-native speakers. Non-native speakers, however, benefited from visual clear speech information, but did not reliably integrate the audio and visual information streams. Taken together, these results suggest that one's language experience plays a role in one's ability to fully utilize multisensory information.

From the possible effect of language experience on speech perception, the current Research Topic also questions the influence of cultural differences on the processing of multisensory information. Zhang et al. compared native Japanese speakers (from Tokyo) to Cantonese learners of Japanese (from Hong Kong) in judging the naturalness of prosodic matching and mismatching stimuli in audio-only and audio-visual modalities. Past research suggests that Cantonese speakers reliably use visual speech cues (Burnham et al., 2022), while Japanese speakers might do so to a lesser degree than other languages (Sekiyama and Tohkura, 1991). The data revealed that both native speakers and learners of Japanese (i.e., native Cantonese speakers) demonstrated minimal integration of visual cues overall, but were more likely to use both audio and visual streams when in mismatched conditions.

Multisensory speech processing continues to be explored in terms of audio-visual processing, yet research has lagged in the integration of haptic information, particularly with regards to neurophysiology. Hansmann et al. breach that gap through investigating tactile sensory input via small air puffs (aerotactile). They provide the first EEG study to compare the behavioral and neurophysiological impact of a unimodal sensory stream (audio-only), to bimodal sensory streams (audio-visual; audio-aerotactile), and a trimodal sensory stream (audio-visual-aerotactile). The behavioral measure revealed an interaction between audio quality (signal-to-noise ratios of −8, −14, −20) and modality, such that as the quality of the auditory signal deteriorated, reliance on the visual modality increased. No effect of tactile information was found. Meanwhile, the EEG results supported previous research in finding processing advantages following exposure to congruent visual information, but not tactile information. To date the impact of aerotactile information in perception has been small (Derrick et al., 2019a,b), suggesting that its utility in speech perception may be revealed when the other information streams in the environment are not able to be used due to degradation of those signals. Thus, in environments rich with auditory and visual sources of information, reliance on additional sensory streams may not be necessary until the information available from those steams becomes salient due to environmental and situational factors, similar to how Sumby and Pollack (1954) originally demonstrated that reliance on information from the visual stream increases in more deleterious hearing conditions.

Notwithstanding, when speech is processed, multiple factors may influence how it is perceived. In a critical review of the literature, Dorsi et al. propose that multisensory information plays a dominant role in speech perception, as compared to lexical information. Their argument lies on evidence that: (1) multisensory information is processed faster at both neurophysiological and behavioral levels; (2) multisensory information influences pre-lexical (sublexical) speech units, which serve to inform the greater lexical unit while impacting interconnected neural systems; (3) multisensory information may be involved in the formation of some lexical information via the sound of a word and its meaning (sound symbolism). Their view, if correct, has implications to not only models of speech perception, but clinical applications for individuals with aphasia or those who have undergone cochlear implants.

In conclusion, the papers featured in this Research Topic provide new insights into multisensory speech perception. The integration of speech information from multiple sensory sources may not be absolute, but instead may be context dependent, varying with language, and language experience (Zeng et al.; Zhang et al.). The research also suggests that reliance on multiple sensory sources may depend on the degree to which information available from any singular source is degraded (Hansmann et al.). Yet, multisensory processing of speech may nonetheless play a primary role in speech perception (Dorsi et al.).

## Author contributions

KS: Writing—original draft, Writing—review & editing. KN: Writing—review & editing. JD: Writing—review & editing.
